# Choosing Therapy for Moderate to Severe Crohn’s Disease

**DOI:** 10.1093/jcag/gwad023

**Published:** 2023-09-22

**Authors:** Malcolm Irani, Bincy Abraham

**Affiliations:** Division of Gastroenterology, Lynda K and David M Underwood Center for Digestive Disorders, Houston Methodist Hospital, 6550 Fannin Street, Smith 1201, Houston, TX 77030, USA; Division of Gastroenterology, Lynda K and David M Underwood Center for Digestive Disorders, Houston Methodist Hospital, 6550 Fannin Street, Smith 1201, Houston, TX 77030, USA

**Keywords:** choosing, therapy, moderate, severe, Crohn’s disease

## Abstract

The availability of approved therapies for Crohn’s disease has significantly increased over the past decade. To choose the appropriate therapy for the patient, ideally head to head studies, and data on positioning could help the provider individualize the decision. Due to the paucity of head-to-head trial data, we turn to network meta-analysis and real-world studies to help guide our treatment choices. Ultimately, the best approach is to consider each patient on an individual basis, taking into consideration the characteristics of their disease, individual risk factors, extra-intestinal manifestations, co-morbid conditions, patient age, cost, and personal preferences. In this review, we summarize the evidence comparing biologic as well as small molecule therapies for the treatment of moderate-to-severe Crohn’s disease. We have summarized the evidence in relation to factors such as efficacy, fistulizing disease, pregnancy, infection risk, and co-existing conditions.

## Introduction

Within the past decade, the number of available therapies to treat Crohn’s disease (CD) significantly increased. Selecting the appropriate therapy, from many options, for patients can be a difficult task. Variability of individual factors, limited data regarding head-to-head trials, and limited generalizability from clinic trial data to specific populations make evidence-based decisions challenging. In addition to ongoing clinical trials, network meta-analysis and real-world studies have helped shed light on this complex problem. Additionally, there are other factors to consider when deciding on appropriate therapy including patient preferences, comorbidities, disease phenotype, inflammatory markers, genetics, and cost.

Treatment goals for CD have also evolved with the new therapeutics. Long-term goals have shifted from symptomatic remission to deep remission, which incorporates both clinical and endoscopic endpoints.^1^ We now use objective markers such as fecal calprotectin, C-reactive protein, and non-invasive modalities such as intestinal ultrasound and magnetic resonance enterography to guide our therapeutic goals.^1^ Forgoing the traditional approach of “step-up” therapy whereby a patient is started on a less toxic therapy prior to initiating biologic medications, growing evidence supports the early use of anti-TNF and biologic medication in the treatment of CD.^2^ This approach has been shown to achieve higher rates of clinical remission and decrease the use of corticosteroids.^3,4^

In this review, we will summarize the current evidence of different treatment strategies and explore the considerations when choosing a therapy for CD.

## Patient factors

It is important to individualize treatment and take patient factors into consideration before choosing a therapy. These factors include patient preference, mode of administration (intravenous vs. subcutaneous vs. oral), medication cost, age, history of malignancy or comorbid conditions, pregnancy for women of childbearing age, pharmacokinetics (body mass index, male sex, high C-reactive protein, low albumin), as well as history of immunogenicity. Patients should feel comfortable with these factors as non-adherence is associated with increased disease activity, mortality, need for surgery, and development of anti-drug antibodies.^5–9^

## Adverse effects of advanced therapies

When choosing a therapy, consider the adverse events for each class of medication to avoid potential complications and side effects. Anti-tumour necrosis factor (anti-TNF)-α agents have been associated with a broad range of infections, including tuberculosis and opportunistic infections.^10^ It is recommended that all patients undergo screening for Hepatitis B and well as tuberculosis before starting this class of medication, as both can be reactivated after treatment initiation.^11^ A positive tuberculosis QuantiFERON test necessitates a chest radiograph to rule out active infection. If active infection is found, guidelines suggest a minimum of 2 weeks of therapy prior to initiation of immunosuppressive agents in coordination with an infectious disease specialist.^12^ Rare cases of optic neuritis, exacerbation of central nervous system disorders, and exacerbation of heart failure have also been described with anti-TNF therapy.^13,14^ For this reason, anti-TNFs are contraindicated in patients with New York Heart Association Functional Class III/IV heart failure and should be avoided in patients with demyelinating disorders.

Regarding malignancy, thiopurine monotherapy or anti-TNF monotherapy is associated with a small risk of lymphoma.^15^ When used in combination, some papers report an increased risk compared to monotherapy.^16–18^ This has not been seen with ustekinumab or vedolizumab.^19–21^ Furthermore, recent data suggest that ustekinumab and vedolizumab do not pose an increased risk of new or recurrent cancer in patients with a previous history of malignancy.^22^ These agents may be preferred in patients at high risk or with a history of malignancy.

The newest medication approved for the treatment of moderate to severe CD is the janus-kinase (JAKi) inhibitor, upadacitinib.^23,24^ This class of medication has a black box warning for increased risk of major adverse cardiovascular events, venous thromboembolism, and malignancy. This warning was extrapolated from data involving another JAKi, tofacitinib, in the treatment of patients with rheumatoid arthritis who were 50 years or older and had at least one cardiovascular risk factor.^25^ This class of medication should be avoided in patients with cardiovascular comorbidities and previous history of venous thromboembolism; however, it is important to note that these increased adverse events have not been seen in patients with inflammatory bowel disease to date. Upadacitinib has also been associated with an increased risk of herpes zoster infection.^24,26,27^ This risk can be mitigated with the recombinant zoster vaccine, and should be recommended to all patients prior to initiation of JAKi therapy.^28^

## Co-existing conditions

Co-existing conditions need to be considered before choosing a therapy. Ideally, one should choose a single therapy with efficacy against the co-existing conditions, which will minimize polypharmacy. Table 1 summarizes the advanced therapies in relation to current approved conditions other than CD. Vedolizumab, due to its gut-selective nature by design, is the only advanced therapy that has no other indications outside the gastrointestinal tract; however, that does not preclude its use in patients with extraintestinal manifestations (EIM).^29^ For those EIMs that parallel luminal disease, vedolizumab may be an effective therapy.^30,31^ In patients with EIMs that do not parallel luminal disease (pyoderma gangrenosum, uveitis, central arthropathy), we would favour anti-cytokine therapies compared to gut-selective agents.^32–34^

**Table 1.** Current approved therapies for moderate-severe Crohn’s disease and other approved indications.

Medication	Other approved indications
Anti-TNF	
Infliximab	Rheumatoid arthritis Ankylosing spondylitis Psoriatic arthritis Plaque psoriasis
Adalimumab	Rheumatoid arthritis Juvenile idiopathic arthritis Psoriatic arthritis Ankylosing spondylitis Plaque psoriasis Hidradenitis suppurativa Uveitis
Certolizumab	Rheumatoid arthritis Psoriatic arthritis Ankylosing spondylitis Non-radiographic axial spondylarthritis Plaque psoriasis
Anti-integrin	
Natalizumab	Multiple sclerosis
Vedolizumab	None
Anti-p40	
Ustekinumab	Plaque psoriasis Psoriatic arthritis
Anti-p19	
Risankizumab	Plaque psoriasis Psoriatic arthritis
Janus kinase inhibitor	
Upadacitinib	Rheumatoid arthritis Psoriatic arthritis Ankylosing spondylitis Non-radiographic axial spondyloarthropathies Atopic dermatitis
Tofacitinib	Only approved for UC
Filgotinib	Only approved for UC

## Efficacy

### Biologic naïve patients

Comparing efficacy between advanced therapies is challenging as there has only been one head-to-head trial to date.^35^ The SEAVUE (Safety and Efficacy of Adalimumab Versus Ustekinumab for One Year) study evaluated efficacy and safety of monotherapy with either ustekinumab or adalimumab in biologic-naïve patients with moderately to severely active CD. At week 52, there were no differences in clinical remission between the ustekinumab and adalimumab groups.^35^ Studies suggest a better safety profile of ustekinumab compared to adalimumab.^36^ Together this suggests that ustekinumab may be a preferred first-line biologic in bio-naïve patients due to its similar efficacy and better safety profile, when compared to adalimumab. However, it is also important to know that patients recruited into the study had early onset disease and were bio-naïve. This suggests that early use of biologic therapy may benefit patients regardless of mechanism of action.

We next turn to network meta-analysis and real-life propensity score weighted data to guide our decisions. A recent meta-analysis compared the efficacy of biologic medications in the induction and maintenance of remission for patients with moderate-to-severe CD and showed superiority of anti-TNF therapy in biologic naïve patients.^37^ Combination of infliximab with azathioprine was ranked higher than infliximab monotherapy for both induction and remission of CD.^37^ This difference was largely driven by data from the SONIC trial, which showed higher rates of corticosteroid-free clinical remission at week 26 in patients treated with combination therapy with infliximab and azathioprine vs. infliximab monotherapy.^38^ This effect may be from targeting more than one inflammatory pathway; however, the sub-analysis of these data suggests that this difference was driven by infliximab trough levels.^38^ This suggests that if you start with combination therapy, you may be able to withdraw the immunomodulator if therapeutic levels are achieved, or that you may be able to use therapeutic drug monitoring to achieve your target level of remission.

Infliximab in combination with azathioprine and infliximab monotherapy had the highest rankings for induction of remission based on surface under the cumulative ranking probability.^37^ This was followed by adalimumab, ustekinumab, risankizumab, vedolizumab, and certolizumab pegol.^37^ When these data were analyzed for maintenance of remission, no individual agent was superior, but the highest ranked were infliximab with azathioprine, adalimumab, and infliximab monotherapy.^37^

### Biologic exposed patients

In patients with previous biologic exposure, ustekinumab and risankizumab had the highest odds of inducing remission compared to placebo.^37^ In patients who had previously failed infliximab, adalimumab was associated with higher odds of clinical remission, compared to placebo. This finding was predominately driven from the GAIN trial, in which patients with a primary-non-response to an anti-TNF were excluded.^39^ In bio-exposed patients, vedolizumab shows improved clinical response but no clinical remission compared to placebo.^37^

## Switching therapy within class

### Anti-TNF

A key consideration prior to switching therapies in bio-exposed patients is the concept of primary versus secondary failure. Approximately one-third of patients do not respond to anti-TNF induction therapy (primary non-responders).^40–42^ Additionally, some patients initially respond to therapy but lose response (secondary non-responders) or develop side effects.^43^ The CHOICE trial evaluated effectiveness of a second anti-TNF in patients with CD who did not achieve remission with their initial anti-TNF. Fifty-one percent of patients achieved remission and the probability of maintaining remission was 64% at 24 months.^44^ Anti-TNF was discontinued due to non-response in 54% of patients and partial response in 46% of patients, which would represent an area of caution when interpreting these data.^44^ Patients with a previous partial response trended toward higher remission rates, compared to those with no response, suggesting that the former may be a better candidate for an inter-class switch.^44^ In addition, a significant proportion of patients lost response to the second anti-TNF over time. Varying response rates were reported in patients starting a second anti-TNF after having a primary non-response to their first anti-TNF, which ranges from 11% to 60% at one year.^44–49^ A recent meta-analysis showed that short-term remission rates were higher when the reason for switching was intolerance compared with primary or secondary failure.^50^ One of the largest studies to date did not find a statistical difference between patients who switched due to primary or secondary failure; however, the loss of response in patients who achieved remission with a second anti-TNF was relatively high.^51^ Thus, a subset of patients who may benefit from switching to another anti-TNF after primary non-response. This suggests that primary failure is not a class-effect phenomenon. An inter-class switch may be more efficacious in those who have failed due to secondary loss of response or intolerance.

Another important consideration with a second anti-TNF is the risk of developing anti-drug antibodies (ADA). Patients who have developed ADA to a prior anti-TNF are at increased risk of developing ADA with their subsequent anti-TNF.^52–54^ In a recent randomized controlled trial (RCT), patients with ADA to anti-TNF monotherapy were randomized to receive anti-TNF monotherapy or in combination with azathioprine. Rates of undetectable trough concentrations with ADA and clinical failure were significantly higher in the monotherapy compared to combination group.^52^ Immunomodulators have shown effectiveness in overcoming as well as prevention of ADA, and should be considered in patients with previous ADA.^54^ Development of immunogenicity to biologics other than anti-TNF is rare, and combination of an immunomodulator with vedolizumab or ustekinumab is not recommended.^55,56^

### IL 12/23

Risankizumab is an anti-p19 antibody that selectively inhibits IL-23, which shares a common target with ustekinumab (anti-p40), which blocks IL-12 and 23.^57–59^ Initially, there were concerns that risankizumab may not be effective in patients who had failed to achieve remission with ustekinumab, due to their shared mechanism of action. The clinical trials for efficacy of risankizumab in CD included patients who failed ustekinumab.^57^ The posthoc analysis showed that patients who were treated with risankizumab after ustekinumab failure had higher rates of clinical remission compared to placebo.^57^ There were also higher rates of endoscopic response in the induction phase compared to placebo.^57^ These effects were also seen in maintenance trials.^59^ These data suggest that there is a subgroup of patients who may respond to risankizumab that have previously failed ustekinumab therapy.

## Combination biologic and/or small molecule

In patients with severe or refractory disease to multiple medications, treatment options can be limited. There is an interest in the role of combination therapy to gain a higher level of response. This may be important for patients who have had a partial response to a certain therapy or have concomitant rheumatological or dermatological disorders which may benefit from targeting an alternative immune pathway.^60^ A recent retrospective study looked at combination therapies with either dual biologic or biologic and small molecule therapies in patients with CD and found higher rates of clinical remission post-combination therapy compared with pre-combination therapy. ^60^ Combination therapy also had significantly more patients in endoscopic remission. Similar findings were reported in systematic reviews that examined safety and efficacy of combination therapy.^61–63^ This approach to therapy should be considered in patients who have concomitant rheumatologic or dermatologic conditions, extra-intestinal manifestations not responsive to monotherapy, severe refractory disease, or those that have had partial improvement to one biologic or small molecule.

## Fistulizing Crohn’s disease

Fistulizing Crohn’s disease (FCD) can be particularly challenging in the management of CD. The optimal treatment strategies involve a multidisciplinary approach with medical and surgical management. Infliximab is the only biologic with proven efficacy in RCTs for FCD.^64,65^ Infliximab showed 46% complete response rate compared to 13% in placebo.^65^ When infliximab was investigated as maintenance therapy, the initial response was 67% at week 14; however, this dropped to 36% at week 54.^64^ This suggests that approximately half of patients who initially respond to infliximab induction may lose responsiveness. Although fistula healing has not been a primary endpoint in RCTs involving adalimumab, several RCTs have investigated this as a secondary endpoint.^42,66^ In the CHARM study, fistula closure was higher in patients treated with adalimumab compared to placebo at weeks 26 and 56.^42^ No major differences have been observed in the efficacy of fistula healing between infliximab and adalimumab in real-world retrospective studies.^67,68^ Of the anti-TNF agents, certolizumab has the least evidence to support its use in FCD. The clinical trials for certolizumab in the treatment of CD showed no difference in fistula healing between patients treated with certolizumab versus placebo at weeks 6 and 26.^41,69^ Due to these poor results, there are no recommendations for the use of certolizumab in the treatment of FCD.^70–72^

The efficacy of combination of an immunomodulator with an anti-TNF has not been evaluated as a primary endpoint in an RCT for FCD, therefore, data regarding its utility is derived from sub-analysis of RCTs. At this time, results are mixed with some retrospective analyses showing higher rates of fistula healing compared to ant-TNF monotherapy, while others show no difference.^73–75^

A recent meta-analysis examined patients with FCD who were treated with ustekinumab and showed fistula response rates of 41%, 39.7%, and 55.9% at weeks 8, 24, and 52, respectively.^76^ Another meta-analysis reported clinical response rates as high as 44% at 6 months and 53.9% at 1 year.^77^ Like ustekinumab, the data regarding the efficacy of vedolizumab has been mainly assessed through subgroup analysis. A recent meta-analysis of four studies in which 87% of patients had failed anti-TNF therapy, showed fistula healing rates of 27.6% in patients treated with vedolizumab.^78^ A recent RCT did not show any benefit from an additional dose of vedolizumab to the standard regimen for fistula healing.^79^

Risankizumab and upadacitinib may have a role in the treatment of FCD; however further studies are needed. Overall, anti-TNFs are the preferred therapy for FCD. In those patients who have failed anti-TNF therapy, both ustekinumab and vedolizumab have shown to be efficacious as second-line biologics.

## Pregnancy

It is critical to consider an individual’s reproductive status prior to choosing a CD therapy. Both maternal and fetal outcomes are improved when women are in remission prior to and during pregnancy.^80^ Disease relapse during pregnancy increases the risks of low birth weight, low gestational age, preterm delivery, spontaneous abortion, and stillbirth. ^81^

### Corticosteroids

The Pregnancy in Inflammatory Bowel Disease and Neonatal Outcome registry (PIANO) did not show an increased risk of gestational diabetes and low birth weight in patients taking corticosteroids; however, other studies have reported a trend toward increased risk of infant infections and preterm birth after steroid exposure.^80,82^ Corticosteroid use was associated with increased risk of low birth weight, preterm birth, infection, and intrauterine growth retardation when given in the second and third trimesters.^82^ Initially, there were concerns for increased risk of cleft lip and palate during the first trimester; however, larger studies have not shown an increased risk.^83^

### Immunomodulators

Existing studies support the safety of azathioprine and mercaptopurine during pregnancy. In the PIANO registry, there was no increased risk of congenital abnormalities or pregnancy complications in patients taking these medications compared to the general population.^80,84^ It is recommended to continue thiopurine monotherapy during pregnancy to maintain remission.

Methotrexate, on the other hand, is a folic acid analog and has shown increased risk of congenital abnormalities and spontaneous abortions. It is recommended that women who want to become pregnant stop methotrexate at least 3 months prior to conception and supplement with high-dose folic acid.^85^ Methotrexate should also be avoided in breastfeeding.

### Biologics

Several registries and meta-analysis support the safety of anti-TNF therapy during pregnancy, with no increased risk of preterm birth, spontaneous abortions, or congenital abnormalities compared to the general population.^86,87^ Certolizumab is the only anti-TNF not actively transferred to the placenta.^86^ Although there is transfer of both adalimumab and infliximab, studies have shown no increased risk of childhood infections, hospitalizations, or antibiotic use in children exposed to anti-TNFs in utero.^88^ Currently, it is recommended that children exposed to anti-TNF avoid live vaccinations for the first 6months of life.^89^ The use of combination therapy with a thiopurine has also been evaluated in the PIANO registry with no increased risk of adverse maternal or fetal outcomes.^80^ Vedolizumab and ustekinumab are also safe to use in pregnancy without increased risk of fetal or maternal complications.^80,90,91^

At this time, there are not enough clinical study data to support the use of risankizumab in pregnant women.^92^ However, it is an IgG monoclonal antibody and likely behaves similarly to all other biologics used in CD suggesting safety in pregnancy and breastfeeding. In contrast, upadacitinib is not preferred in pregnant or breastfeeding women.^26,27^ In animal studies, upadacitinib administration resulted in a dose-dependent increased risk of fetal malformations and post-implantation loss.^93^

## Conclusions

The number of biologic and small molecule therapies available for the treatment of CD continues to grow. Several studies have individually assessed the efficacy and safety of these therapies, but the lack of head-to-head trials can make medication selection difficult. We have summarized our recommendations based on specific conditions in Table 2.

**Table 2.** Author recommendations for therapy choice in different situations.

Situation	Recommendation
Biologic naïve	UST, RZA, VDZ, anti-TNF
Anti-TNF failure: primary non-response	UST > VDZ, UPA, RZA
Anti-TNF failure: secondary failure	Consider anti-TNF+/− immunomodulator, UST > VDZ, RZA, UPA
VDZ failure	Anti-TNF, UPA, UST, RZA
UST failure	Anti-TNF, UPA, VDZ, consider RZA
RZA failure	Anti-TNF, UPA, VDZ, consider UST
Demyelinating disease	Avoid anti-TNF
Congestive heart failure	Avoid anti-TNF and UPA
SLE	Avoid anti-TNF
Frequent infections	Avoid anti-TNFs and UPA
IBD associated EIMs	UST,RZA, UPA, anti-TNF > VDZ
Fistulizing disease	First line anti-TNF, consider UST or VDZ as second line
Elderly	UST, VDZ, RZA
Pregnancy	Avoid UPA, lack of data for RZA
History of malignancy	VDZ, UST, RZA, consider anti-TNF

Abbreviations: anti-TNF, anti-tumour necrosis factor inhibitors; RZA, risankizumab; UPA, upadacitinib; UST, ustekinumab; VDZ, vedolizumab.

The best approach is to consider each patient on an individual basis, taking into consideration the characteristics of their disease, individual risk factors, extra-intestinal manifestations, co-morbid conditions, patient age, cost, and personal preferences.

## Data Availability

No new data or analysis was generated for this work.
